# Developing computable sustainable urbanization science: interdisciplinary perspective

**DOI:** 10.1007/s43762-022-00048-9

**Published:** 2022-06-20

**Authors:** Mingxing Chen, Liangkan Chen, Yang Li, Yue Xian

**Affiliations:** 1 Institute of Geographic Sciences and Natural Resources Research, Key Laboratory of Regional Sustainable Development Modeling, Chinese Academy of Sciences, Beijing, 100101 China; 2 University of Chinese Academy of Sciences, Beijing, 100049 China; 3 China Center for Modernization Research, Chinese Academy of Sciences, Beijing, 100190 China

**Keywords:** Global urbanization, Interdisciplinary, Scientific computation, Sustainable

## Abstract

In this era of drastic global change, the Anthropocene, carbon neutrality and sustainable development have become common twenty-first century human challenges and goals. Large-scale urbanization is indicative of human activities and provides an important impetus for environmental changes; therefore, cities have become an important stage in which to promote a more sustainable future development of human society. However, current researchers study urbanization issues based on the perspectives and tools of their respective disciplines; therefore, a holistic and comprehensive understanding of urbanization is lacking due to the insufficient integration of multidisciplinary study perspectives. We explored the construction of interdisciplinary computable sustainable urbanization and introduces a conceptual framework for interdisciplinary urbanization, as scientific computing supports and integrates the natural sciences and humanities to simulate urban evolution and further observe, explain, and optimize human and environment interactions in urban areas. We advocated for the establishment of major international research programs and organizations in the field of sustainable urbanization, and the cultivation of talented young professionals with broad-ranging interdisciplinary interests. Expectantly, we hope a livable planet in the Anthropocene era could be created by developing sustainable urbanization and achieving carbon neutrality.

## Changing Earth in the Anthropocene era

Since 2021, extreme disasters have regularly occurred worldwide; the potential consequences of cross-scale systemic environmental risks with global impacts are increasing (Keys et al., 2019). An extreme heat wave affected much of western North America from late June through mid-July 2021, resulting in record high temperatures throughout the region, including record highs exceeding 40 °C in many places in the United States and record highs of 49.6 °C in Canada. Days of high temperatures sparked numerous extensive wildfires, damaged road and rail infrastructure, forced business closures, caused extensive damage to crop and resulted in approximately 600 excess deaths across the region (Popovich & Choi-Schagrin, 2021). Extreme heavy rainfall in central and western Europe triggered rare severe floods, which caused nearly 200 deaths in Germany; the worst-affected regions were Rhineland-Palatinate and North Rhine-Westphalia (Fitzgerald et al., 2021). The heavy rainfall in China’s Henan Province from 17 July to 31 July 2021 affected a total of 150 counties, 1663 townships and 145.32 million people, with 302 people dead and 50 declared missing (Liu et al., 2021). A series of outbreaks, such as the COVID-19 pandemic and geo-environmental events, further aggravated and worsened the state of global sustainable development.

The Earth is in an era of significant changes, the causes of which are primarily driven by human activities that have occurred since the Industrial Revolution and that include large-scale urbanization and industrialization and various economic and social activities, such as manufacturing and the adoption of sophisticated lifestyles (Steffen et al., 2015). The correlation between complex changes and systems has been recognized (Liu et al., 2018; Cumming et al., 2014). Changes in atmospheric composition and land cover caused by continuous human activities, as well as their interactions and feedbacks, will have important impacts on the Earth (Yue et al., 2020; Steffen et al., 2018; Gruber & Galloway, 2008). Human-induced greenhouse gas emissions affect global climate change through atmospheric circulation, resulting in global warming (McMichael, 2013), and every 0.5 °C increase in temperature will lead to an increase in the intensity and frequency of global extreme heat, heavy rainfall, and drought events (Li et al., 2021). The world has been undergoing a marked process of urbanization, especially in developing countries, in recent years (Sun et al., 2020). City changes in population size, production levels, lifestyles, greenhouse gas and pollution emissions, etc. cause cities to not only suffer from the influence of global climate change but also bear the indisputable responsibility for global climate change (Rosenzweig et al., 2010).

In this era of drastic changes, several new concepts have been proposed. The Anthropocene, carbon neutrality and sustainable development are the most important among them, and they represent the common challenges and development goals faced by humankind in the twenty-first century. Based on a brief review of these three related concepts, this article proposes that human activities represented by large-scale urbanization are the key drivers of changes to the Earth and that there is an urgent need to dismantle the barrier between disciplines, especially the gap between natural science and human and social sciences. To promote the sustainable development of humankind and prioritize ecological and environmental issues, we can improve sustainable urbanization by utilizing scientific computing as an important technical method, advocate for the establishment of major international research projects and organizations, and vigorously cultivate talented young individuals working in interdisciplinary fields.

### Anthropocene

The concept of the “Anthropocene” was proposed for the current geological epoch in 2000 by Dutch atmospheric chemist Paul J. Crutzen, who was awarded the Nobel Prize in Chemistry in 1995 (Crutzen, 2002a). In May 2019, the 29 members of the Anthropocene Working Group (AWG) voted in favor of a starting date in the mid-twentieth century, stating that in this period “a rapid rising human population accelerated the pace of industrial production and intense human activities made an irreversible and huge impact on the Earth”; therefore, it seems appropriate to assign the term ‘Anthropocene’ to a new, human-dominated geological epoch (Crutzen, 2002b). Since the middle of the first industrial revolution, the population has increased from 1 billion in 1800 to 7.6 billion in 2020, with a growth rate as high as 300 million per 10 years; this growth rate has been increasing, and Earth’s population has far exceeded its threshold (Godwin, 1820).

As a result of the strong influence of human activities, the main forces changing systems on the Earth have shifted from natural forces such as freeze and thaw cycles, water power, wind power, and gravity to humankind’s influence. For more than two hundred years, rapid population growth has accelerated the pace of human activities such as industrial production and has exhausted the fossil fuels formed over millions of years, leading to global warming, rising sea levels, and reducing biodiversity. Human forces have posed enormous systemic risks to Earth; they are irreversible and will influence on the Earth’s systems for the next ten thousand years (Tin et al., 2009).

### Carbon neutrality

It is commonly agreed that global warming is a prominent consequence of climate change caused by human activities. In the past ten years, highest temperature on record occurred, and apocalyptic fires, floods, droughts, storms and rising sea levels have become the new normal. Over 170 countries signed “The Paris Agreement” on 22 April 2016 at the UN headquarters to limit the increase in global warming and improve adaptative abilities to combat the adverse impacts of climate change. The goal of the agreement is to limit global warming to well below 2, preferably to 1.5 °C, compared to preindustrial levels, through country-level actions to reduce carbon emissions (Falkner, 2016). To achieve this long-term temperature goal, countries aim to reach global greenhouse gas emissions peaks as soon as possible, and reaching carbon neutrality by the mid-twenty-first century is essential. The transition from carbon peak to carbon neutrality is a new goal under the 1.5-degree temperature control target on climate change, which is the core issue of “The Paris Agreement” for the foreseeable future.

Carbon neutrality is a state of net-zero carbon dioxide emissions achieved by balancing emissions of carbon dioxide with its removal or eliminating CO_2_ emissions in amounts equal to what human society produces. It is the only way to cope with climate change and is an embodiment of the desire for human beings to engage in development that is compatible with the global environment. As of June 2021, approximately 137 countries around the world had committed to carbon neutrality and set timeline targets; 131 countries are aiming for approximately 2050 as their target date (CAT, 2021). In addition, 733 cities, 31 regions, 3067 businesses, 173 of the largest investors, and 622 Higher Education Institutions have joined the Race to Zero Campaign and committed to achieving carbon neutrality by 2050 (UNCC, 2019). Countries that have made a commitment to carbon neutrality should layout a low-carbon transition development roadmap guided by a 1.5 °C target and strictly control carbon emissions. However, the realization of the global carbon emission target requires the active response and cogent actions of more countries and international organizations.

### Sustainable development goals

The Sustainable Development Summit of adopted the 2030 Development Agenda titled “Transforming our World: The 2030 Agenda for Sustainable Development” and outlines 17 Sustainable Development Goals (SDGs) and the associated 169 targets and 232 indicators, which are integrated and indivisible and balance the three dimensions: economic, social and environmental (UN, 2015). The goals and targets create a systemic blueprint for the sustainable development of humanity and the planet over the next 15 years by stimulating action in areas of critical importance and indicating the direction for global development and international cooperation (Costanza et al., 2016). A deepening knowledge of sustainable development has led to a global development consensus that the pursuit of social and economic prosperity must occur with respect to the constraints of natural resources. SDGs stress the realization of sustainable and coordinated development among society, the economy, and the environment and significantly elevate the importance of environmental goals (Griggs et al., 2013). Approximately 3.5 billion people (55%) now live in cities around the world, and rapid urbanization has created many problems and challenges.

The sustainable development of urban areas is crucial to the current sustainable development of human society and is also the key to achieving all SDGs. Focusing on human development, resource consumption and pollution emissions, SDG 11 aims to “make cities and human settlements inclusive, safe, resilient and sustainable”, identifies the coordinated development problems between urbanization and the ecological environment, and promotes the sustainable development of global urbanization (Huovila et al., 2019). At present, 150 countries around the world have formulated corresponding national urban plans, and nearly half of them are in the implementation stage. Achievement of these goals helps global cities develop in a safer, more inclusive, and more sustainable manner.

### Nexus among the Anthropocene, carbon neutrality and SDGs

In the Anthropocene, human activities have a huge impact on the dynamic changes in the global carbon cycle, in terms of land use change, large-scale urbanization, and further reshaping of the Earth’s surface (Steffen et al., 2018; Song et al., 2018). The huge exploitation of fossil fuels has caused the concentration of greenhouse gases in the atmosphere to rise rapidly, and the greenhouse effects have further accelerated global change (Schuur et al., 2015; Zhou et al., 2021). The carbon cycle processes and the urbanization process both are important processes on the Earth’s surface, which reflect the interaction between man and land. In-depth exploration of the interactions, impact mechanisms and implementation pathways of climate change, carbon neutrality and sustainable urbanization, is an important content for the comprehensive understanding of multi-layer coupling and interfacial processes in the Earth’s surface system, and helps to promote the synergistic effects of multiple objectives such as SDGs, carbon neutrality and sustainable urbanization (Canadell et al., 2010; Chen et al., 2021). It is important to recognize that human activities represented by large-scale urbanization are important causes of climate change, including greenhouse gas emissions (Khoshnevis Yazdi & Golestani Dariani, 2019), including the impact of urbanization on natural ecosystem elements such as surface vegetation and hydrology (Falkowski et al., 2000; Dangendorf et al., 2019). A systematic understanding the nexus among the Anthropocene, carbon neutrality and sustainable urbanization, is the key to human adaptation and adaptation strategies that are actively responding to climate change, and whether carbon neutrality goals can be achieved as scheduled.

## Large-scale urbanization is a major driver of global change

Urbanization is the most significant human activity currently occurring on the Earth’s surface. In recent decades, the world has experienced large-scale and rapidly expanding urbanization (Chen et al., 2014, 2021). This rapid urbanization has been accompanied by concentrated growth in urban population, dramatic changes in land use, and soaring carbon emissions from the massive fossil energy burning.

### Rapid urban population growth

**Fig. 1:**
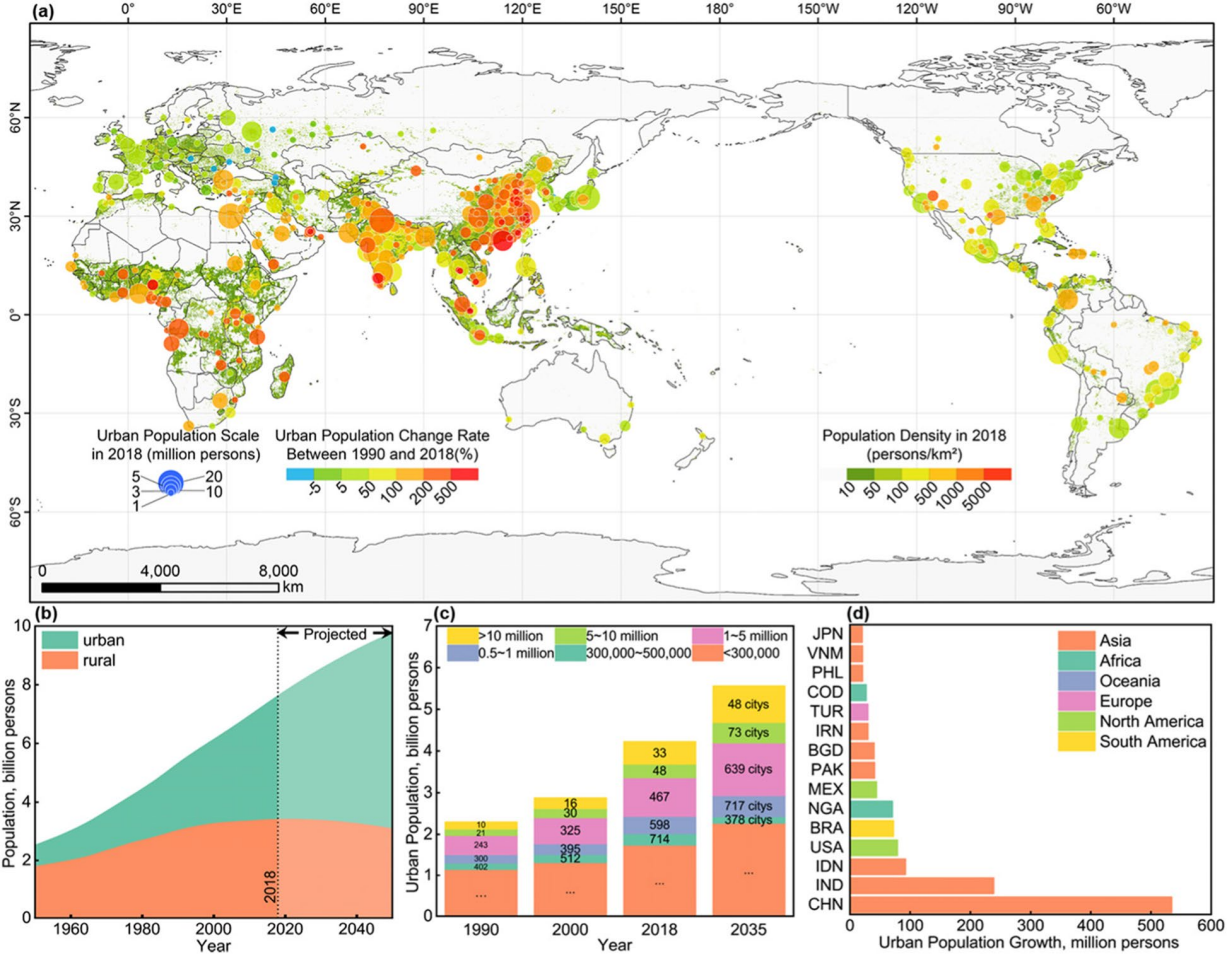
Spatio-temporal distribution of global urban population in 1990-2018. **a** Spatio-temporal distribution of population growth in major cities with population over 1 million in the world, 1990-2018; **b** Total, urban, and rural population, 1950-2050; **c** Population and number of cities of the world, by size class of urban settlement, 1990, 2020, 2018, 2035; **d** Top 15 countries with the largest population growth, 1990-2018)

The impact intensity of human activities increases with the rapid growth of the global population; large-scale urbanization has increased the proportion of the global urban population from 29.6% in 1950 to 55.28% in 2018 and consequently accounts for most of the dramatic population changes and impacts of humankind. According to UN reports (UN, 2018), as of 2020, urban areas accommodated approximately 4.38 billion people, and that number will reach 6.68 billion by 2050; urbanization will continue to play a major role in human activities (Fig. 1b).

During 1990-2018 (Fig. 1a), the global urban population increased from 2.29 billion to 4.22 billion, with Asia, Africa, and North America as hotspots; of these, China, India, and sub-Saharan countries are the most prominent. However, the urban population growth rate in developed countries such as Japan, Korea, and European countries is relatively low, and some cities in Eastern Europe have been experiencing urban shrinkage.

The continuous expansion of the urban population has also accelerated the incubation of large-scale global cities (Fig. 1c). In 1990-2018, medium-sized cities with populations between 1 and 5 million increased from 243 to 467, large cities with populations between 5 and 10 million increased from 21 to 48, and megacities with populations greater than 10 million increased from 10 to 33; 18 more large cities and 13 more megacities will be added by 2030. For country-level populations (Fig. 1d), urban population growth was mainly concentrated in China and India, with increases of 527 billion (27.3% of world increase) and 238 billion (12.4%), respectively, followed by Indonesia, the United States, Brazil and Nigeria. Developing countries are experiencing accelerating urbanization, and booming industries and services can provide many employment opportunities for the newly increasing urban population and encourage more rural residents to migrate to cities.

### Dramatic increase in built-up area

The global impervious surface area (ISA) reached 797,076 km^2^ in 2018, which is almost 2.5 times greater than that in 1990 (Gong et al., 2020). The global ISA showed a rapid growth pattern from 1990 to 2015, with an average annual growth rate of 3.0% in 1990-2010 and 4.4% in 2010-2015, while the speed of expansion of the global impervious surface area began to slow down after 2015.

**Fig. 2:**
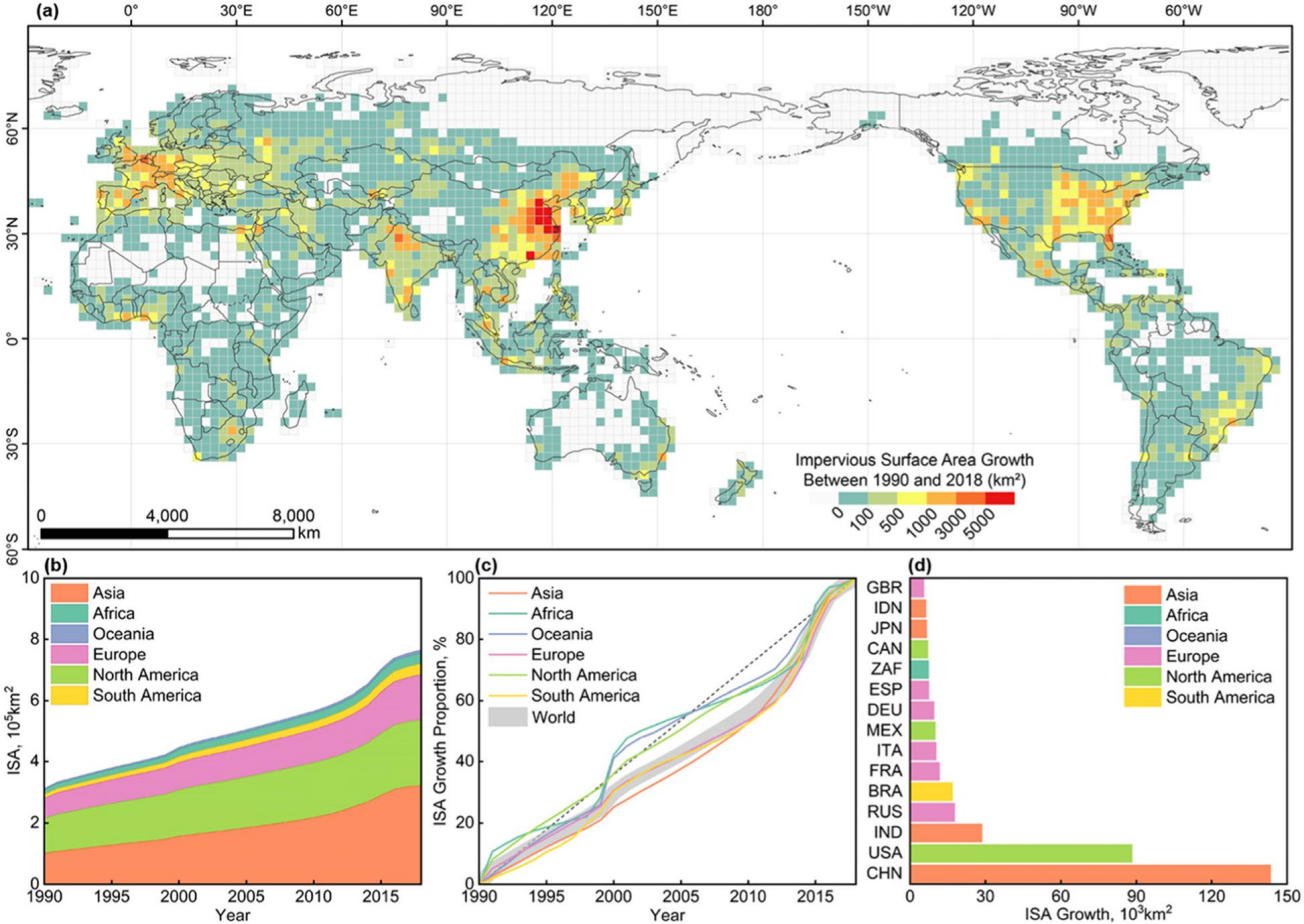
Spatio-temporal distribution of global artificial impervious area growth, 1990-2018. **a** Spatio-temporal distribution of global artificial impervious area growth, 1990-2018; **b** The growth trend of artificial impervious area by continent, 1990-2018; **c** Cumulative growth proportion of ISA, 1990-2018; **d** Top 15 countries with the largest ISA growth, 1990-2018)

Spatially, ISA growth was mainly concentrated in the eastern coastal areas of China, followed by significant growth in the eastern United States, India, western Europe (France, Germany, Italy) and the eastern coastal areas of Brazil (Fig. 2a). At continental level (Fig. 2b), the growth of ISA during 1990-2018 was faster in Asia and North America than elsewhere, where it was 231,663 km^2^ and 104,412 km^2^ respectively, followed by Europe with an increase of 88,684 km^2^, South America with an increase of 25,262 km^2^, and Africa with an increase of 22,281 km^2^.

Population growth is part of the reason for the rapid growth of artificial impervious areas in East Asia; however, it is worth noting that Africa, a region with significant urban population growth, had relatively slow growth in terms of ISA (Fig. 2c). At the country level (Fig. 2d), China and the United States dominate ISA growth, with mean growth rates of 4663 km^2^ per year and 2894 km^2^ per year, respectively, accounting for 51% of the global impervious surface area growth rate, followed by India, Russia, and Brazil.

### Soaring carbon emissions

**Fig. 3:**
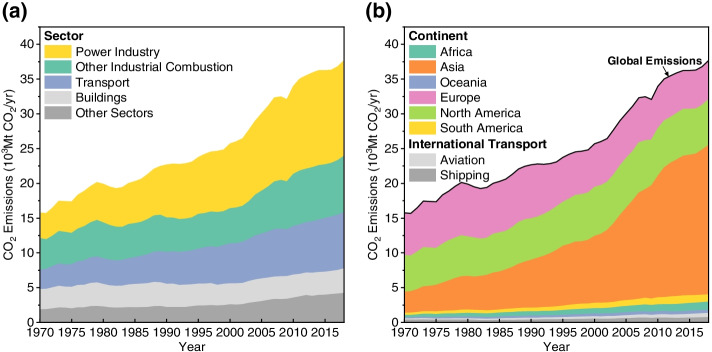
Global CO_2_ emissions, 1970-2018. **a** Global CO_2_ emissions by sector, 1970-2018; **b** Global CO_2_ emissions by continent, 1970-2018)

The global annual CO_2_ emissions increased from 15.76 Gt CO_2_ to 25.71 Gt CO_2_ in 2000, and reached 37.67 Gt CO_2_ in 2018 (Crippa et al., 2020). In this half century, the global carbon emissions increased tremendously and at an accelerated rate. Regarding the global emission structure by sector (Fig. 3a), the power industry was consistently the top source of carbon emissions during 1970-2018, with its proportion increasing from 23% to 36%, an increase of 10.04 Gt CO_2_; it was followed by the industrial and transport sectors. Industrial emissions increased by 3.59 Gt CO_2_, while the share of emissions from the industrial sector dropped from 29% to 22%. Transport sector emissions increased by 5.29 Gt CO_2_, and its share rose from 18% to 21%. Building sector emissions increased by 0.62 Gt CO_2_, and its share dropped from 19% to 9%. The share of emissions from the remaining sectors remained the same.

Rapid urbanization relies on a large amount of fossil energy consumption. Hence, carbon emissions in Asia featured a low initial volume but high growth and a steady and significant increasing trend, especially after the twenty-first century. However, their growth slowed after approximately 2011. Carbon emissions in Africa maintained a stable growth rate and began to rise after 2000. Europe and North America reached their carbon peaks in 1990 and 2007, respectively (Fig. 3b).

Since 1970, China’s carbon emissions have continued to rise, surpassing the European Union in 2003 and the United States in 2005 to become the world’s largest CO_2_ emitter, accounting for 28% of total global CO_2_ emissions in 2018. In addition to China, India’s CO_2_ emissions proportion has gradually increased to 7%. CO_2_ emissions in the United States, the European Union and Russia have reached their peaks. However, regarding to cumulative emissions, the United States accounts for 25% of the world’s total CO_2_ emissions, the European Union accounts for 22%, and China accounts for 13% (Hannah, 2019). Developed countries and regions have basically completed the urbanization process, while urbanization in developing countries is still in progress. There is tremendous demand for infrastructure construction, which still requires growing energy consumption.

## Multiple challenges facing the future urbanization science

At present, there is still a lack of comprehensive scientific understanding regarding sustainable urbanization in the Anthropocene and carbon neutral policy scenarios (Elmqvist et al., 2021). Scientists need to systematically understand the dynamics of urbanization and its impact on global climate change. A deep understanding of sustainable urbanization is helpful to predict its speed, scale, structure, and sustainability accurately and scientifically. In the urbanization process, the feedback and responses of the systems on the Earth to human disturbance are complex and nonlinear in nature and involve not only natural science issues but also complex social science issues (Chen et al., 2010; Tortell, 2020). Thus, the following three challenges are worthy of further attention by scientists.

**Fig. 4:**
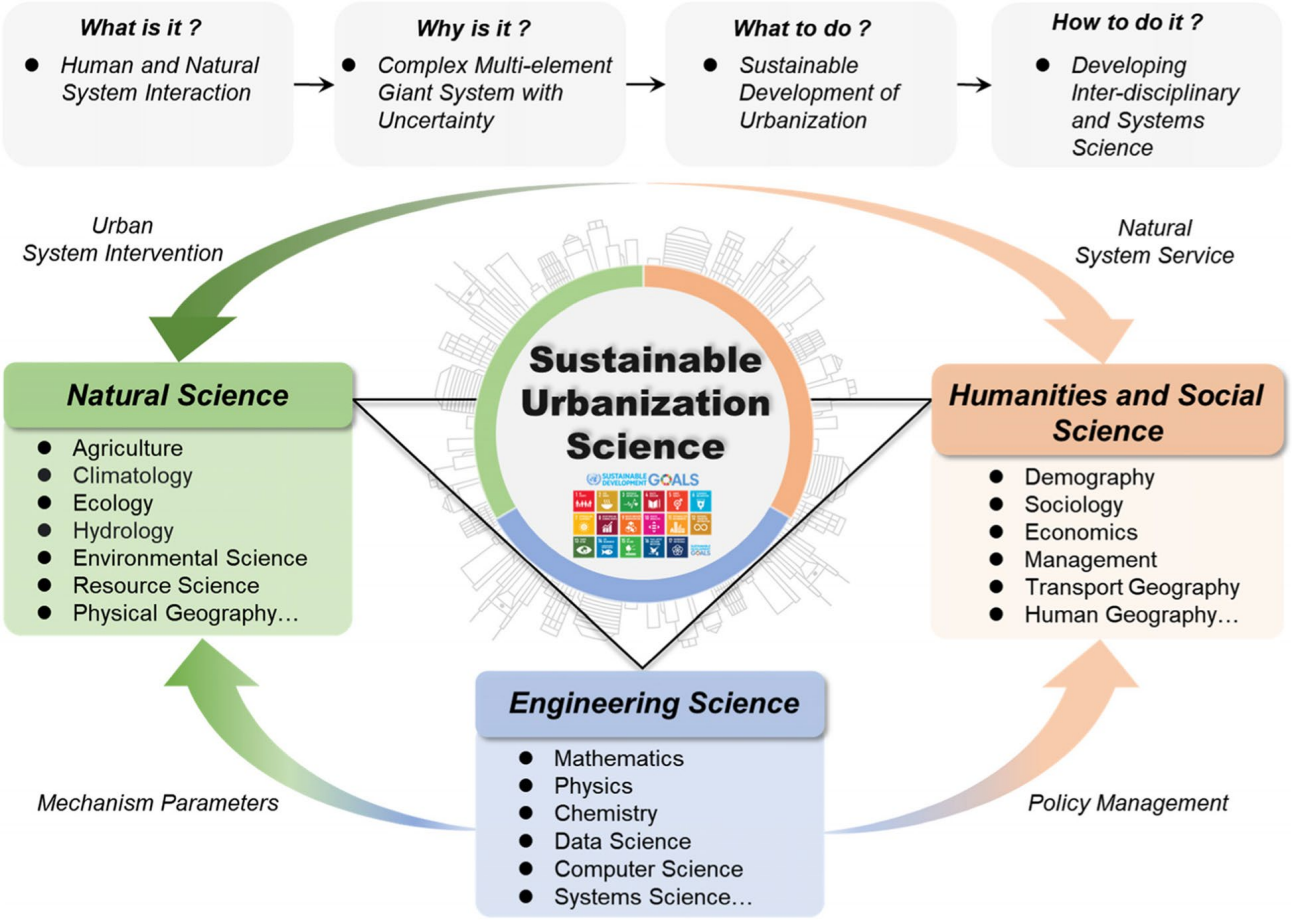
Conceptual inter-disciplinary model of sustainable urbanization science

First, current studies are scattered across multiple disciplines with an intrinsic gap between the natural sciences and humanities; hence, it is difficult to develop an integrated cross-disciplinary research system. As shown in Fig. 4, research on sustainable urbanization involves population, economy, society, ecology, management, and other aspects, thereby attracting the participation of many disciplines, such as Earth sciences, energy sciences, environmental sciences, ecological sciences, economic sciences, and social sciences. Current urbanization research is still fragmented and independent. Lack of diversified integration of disciplines and comprehensive understanding of urbanization leads different disciplines to study urbanization issues based on their own perspectives and methods, resulting in a ‘smorgasbord’ of research results.

Therefore, it has become urgent to further strengthen basic research in related fields, promote interdisciplinary and cross-field academic exchanges and discussions, and deepen common knowledge and mutual understanding. It is necessary to think deeply about filling this gap, that is, to establish a sustainable urbanization science and focus on sustainable urbanization. An increasing number of research teams have realized the necessity and urgency of establishing an urbanization discipline (Acuto et al., 2018; Chen et al., 2019).

Second, due to the lack of basic subject accumulation and technological breakthroughs, it is difficult to meet both the urgent requirements of global sustainable development and the needs of scientific decision-making. In recent years, large-scale urbanization, carbon emissions, and sustainable development relationships have combined into a newly emerging and multidisciplinary field of research (Behera & Dash, 2017; Zhang et al., 2019; Mao et al., 2021); therefore, there are shortcomings in the accumulation of basic subject research compared with other classical subject fields.

Furthermore, due to the substantial variation in the different development stages, economy-social-cultural foundations, institutions and governance systems as well as the natural geographical environment and man-land relationship between the global north and south, it is hard to forge a unified solution or paradigm that can solve the sustainable problems of all countries, which greatly increases the difficulty of providing for the needs of complex scientific decision-making. Given the key role of cities in carbon emissions and sustainable development, there are two main issues that need further and deeper understanding and exposition: one is how to connect to the UN’s SDG strategy, and the other is how the speed, scale, mode, and spatial organization of sustainable urbanization development affect adaptions to new strategies under the new targets of carbon neutrality.

Third, the construction of professional teams and the training of young scientists are far from adequate. At present, the research teams engaged in the Anthropocene, carbon neutrality, and sustainable urbanization research are insufficient, and their knowledge structure is not yet perfect. Today’s talent-cultivating mode is distributed inside individual disciplines, which are vastly unable to meet the needs of new situations and new demands. Therefore, strengthening the training of scientific and technical personnel at all levels is the backbone for this interdisciplinary field and should be a top priority.

## Developing an inter-disciplinary and computable framework for sustainable urbanization

New disciplinary research paradigms of the sustainable urbanization discipline must be developed and implemented to understand our rapidly evolving urbanization processes and to further monitor the impact of measures affecting the habitability of the Earth. The paradigm of computational urbanization research introduces new cross-cutting perspectives and factors through geographic big data. Geographic big data contains multi-modal and rich media that can provide an integrated view of multivariate correlations and causal relationships. (Chang et al., 2014). These data are important for urban management decisions, which often require an understanding of why and how things happen.

**Fig. 5:**
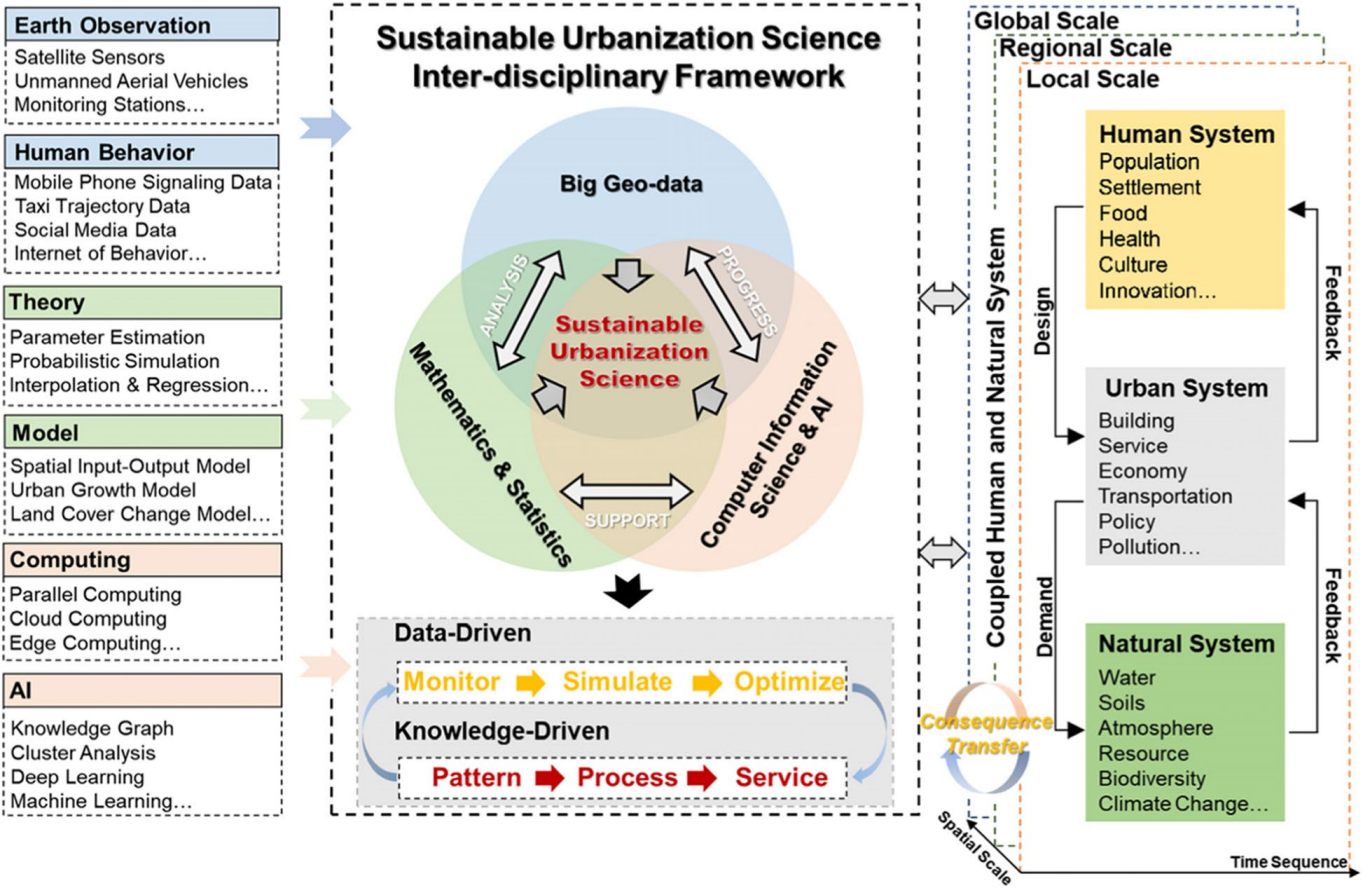
Inter-disciplinary research framework of technical methods in the field of computable sustainable urbanization

First, we advocate for building a sustainable urbanization discipline that uses scientific computing as an important means of supporting technical methods (Fig. 5). In consideration of the Anthropocene-oriented development era, a computable scientific and technological methods system is urgently needed to analyze carbon neutral development targets and the complex characteristics of the objects, methodologies, and multiple scales of sustainable urbanization research. Here, computability mainly comprises the integration of big geospatial data, mathematical and statistical sciences, information science and artificial intelligence.

Big data and geographic information science and technology have considerably improved data acquisition and analysis. Support from technologies such as informatization, digitalization, gridding, cloud computing, remote sensing, and spatial positioning techniques allows us to capture massive amounts of individual information and point-of-interest data in real time for the urbanization process, urban development, and the evolution of human-land relationships (Trevisani & Omodeo, 2021; Wang & Ye, 2018; Guo et al., 2016); we can then launch in-depth analyses on the spatial form, spatial overlay, spatial topology, and spatial networks of geographical entities.

Mathematical and statistical sciences have been promoted intensively for non-classical parameter estimation methods for optimal solutions, such as maximum likelihood estimation, generalized moment estimation, Bayesian estimation, and quantile regression estimation (Zhu et al., 2020). The semi-parametric model combines a parametric model and a nonparametric model, and the model has both parametric variables with known functional forms and nonparametric variables with unknown functional forms, which facilitates the analysis the complex mechanism underlying the bilateral effect between sustainable urbanization and the human-land relationship (Mashhoodi, 2021). The above provides a scientific methodological basis for future predictions.

Machine learning methods such as random forests, logistic regression, and neural network techniques often show superior results in estimating parameters than the traditional information obtained from sample statistics. Various machine learning algorithms that mimic human self-learning have been developed and applied through a combination of artificial intelligence, machine learning, genetic algorithms, and artificial neural networks (Jean et al., 2016; Ye et al., 2021). The combined application of multiple models can form a composite model, which is gradually becoming a trend in technical approaches to simulate urbanization and regional development processes (Reichstein et al., 2019). Although research on artificial intelligence and machine learning in urbanization science is still at its infancy, they are the main directions for future technological and methodological innovation.

Computable urbanization science is a novel paradigm that enables researchers to address sustainable urbanization questions based on correlations and cause-effect relationships across multiple fields of data, in clear contrast to traditional approaches that are difficult to manage. Data-driven sustainable urbanization science will help provide high-quality contributions that lead researchers to explore and develop new theories or models, create new analytical algorithms or platforms, and establish new governance or regulatory decisions that enable data empowerment and expertise creation.

Second, a greater and deeper integration of natural science and social science should be developed. In the era of climate change and the Anthropocene, researchers should study the coupling mechanism of natural elements and human element processes, further understand the profound impact of human activities on the Earth’s surface system, and make breakthroughs in recognition mechanisms and research methods (Wang et al., 2018; Fan, 2018). We should Integrate the comprehensive and intersecting scientific advantages of geography, establish a complex man-land system simulation model with systemically holistic comprehensive approach (Fu, 2017), improve the elaborate simulation capabilities of coupling the “human-land- atmosphere” system, deepen integrated research and applied research for decision making. And further the harmonious development of city and environment, the cross-fertilization development of urbanization and carbon neutrality, to provide scientific basis for sustainable development of watershed, region, country and even the world.

Third, the international science program organization involving the Anthropocene, carbon neutrality and sustainable urbanization should be established, to design major research projects involving interdisciplinary fields and multi-national participation. The International Science Programme (ISP) is an important means for humankind to explore the unknown world, and solve major global issues, as well as a comprehensive expansion of basic research at the frontier of science. It plays a vital underlying role in driving global scientific innovations, addressing common challenges faced by the global human community, and building a global community for shared future of humanity.

Series of multi-circle changes on the Earth’s surface resulting from human influence have become a major multidisciplinary research object. As a human-dominated geospatial process, urbanization has increasingly impacted the Earth’s surface. In the era of the Anthropocene, reasonable and urgent choices to promote sustainable urbanization, achieve the goal of carbon neutrality, and build a habitable Earth, including establishment of the international scientific organization; developing disciplinary development roadmaps on the Anthropocene, carbon neutrality and sustainable urbanization; and integrating scientific research with decision-making applications.

Fourth, vigorous cultivation of cross-integrated young talented individuals is needed. The inheritance of knowledge from generation to generation is the key to scientific development, and it is often the case that the structure is lacking to support the necessary number of cross-integrated individuals, as is the discipline for the sustainable urbanization science. It is necessary to enhance and nurture young talented individuals, create relevant academic subjects or disciplines in universities and scientific institutions, cultivate more young talented individuals with cross-integrated knowledge, and build a sustainable echelon of talented professionals to support the future development of the discipline.

In summary, in the context of the Anthropocene and carbon neutrality, sustainable urbanization is a scientific issue that deserves the attention of scientists in the future, as it is closely related to human production and life. Human activities have an increasing impact on the Earth, and urbanization is the most significant and representative of these activities in geographic space. Therefore, exploring the trajectory of sustainable urbanization contributes equally to the future sustainable development of humanity and the construction of a livable Earth. In recent years, frequent outbreaks of epidemics and extreme weather disasters have converged with urbanization in dangerous ways, and the uncertainty of achieving sustainable urbanization remains a substantial challenge for the scientific community.

## Data Availability

Supplementary data to this article are openly available online at the following URL: The trends of population of urban agglomerations by geographic region can be obtained from World Urbanization Prospects 2018, United Nations. (Source: https://population.un.org/wup/Download/). The Urban land use change datasets are derived from the global artificial impervious area (GAIA). (Source: http://data.ess.tsinghua.edu.cn/gaia.html). The classifies CO_2_ emissions of the world according to the Emissions Database for Global Atmospheric Research (EGDAR) in 2019. (Source: https://edgar.jrc.ec.europa.eu/dataset_ghg60).
